# Prevalence of Ocular Hypertension and Other Risk Factors of Open-Angle Glaucoma Among Middle-Aged Adults in Al-Baha City, Saudi Arabia

**DOI:** 10.7759/cureus.50304

**Published:** 2023-12-11

**Authors:** Mahadi Bashir, Ali H Alghamdi, Suhaib A Alzahrani, Abdullah M Alhajji, Loay Y Al Thobaiti, Khalid A Alzahrani, Ahmed G Alghamdi, Ranin K Alnemari, Abdulaziz H Althobaiti, Roaa S Alzahrani

**Affiliations:** 1 Ophthalmology, Faculty of Medicine, Al Baha University, Al Baha, SAU; 2 General Practice, Taif Health Cluster, Taif, SAU; 3 General Practice, Al-Ahsa Health Cluster, Al-Ahsa, SAU; 4 General Practice, Al-Baha Health Affairs, Al Baha, SAU; 5 College of Medicine, Al Baha University, Al Baha, SAU

**Keywords:** cross-sectional study, al-baha region, glaucoma risk factors, intraocular pressure, ocular hypertension

## Abstract

Background

Ocular hypertension (OHT), defined by elevated intraocular pressure (IOP) beyond standard levels, is a predominant risk factor for initiating and exacerbating glaucoma, a collection of progressive optic neuropathies responsible for irreversible vision loss. Given the profound implications for vision care, it is imperative to elucidate the interplay between OHT and glaucoma for effective clinical management.

Objective

The present study aims to measure IOP levels and identify risk factors associated with glaucoma among middle-aged individuals in Al-Baha City, Saudi Arabia.

Methods

A cross-sectional study was conducted over a six-month span (January-June 2022) in Al-Baha City. The study cohort comprised adults aged 35 and above attending a glaucoma awareness campaign at King Fahad Hospital, Al-Baha. Parameters such as demographics, socioeconomic status, medical and ocular history, and familial history of eye diseases were collated. Initial ophthalmologic assessments and IOP measurements were performed. Statistical analyses utilized Pearson's Chi-square test for nominal variables.

Results

The study encompassed 111 participants, 84 (75.7%) of whom were male, and 75 (67.6%) were of Saudi nationality. Notably, 102 (91.9%) reported no family history of glaucoma, 91 (81.1%) indicated no past medical history and 81 (73.0%) were not on any chronic medications. The mean IOP for participants' right and left eyes fluctuated between 18.2-21.5 mmHg and 18.9-22.1 mmHg, respectively. Factors such as age, gender, family history of glaucoma, past medical history, use of chronic medications, and history of ophthalmic surgeries demonstrated a statistically significant correlation with IOP (p<0.05).

Conclusion

This study highlights a higher prevalence of OHT in females, with several risk factors for OHT and glaucoma identified, such as familial history, vascular diseases, diabetes mellitus, and chronic medication use. Notably, our study did not observe a significant association with age or smoking. These findings emphasize the necessity of regular eye examinations and IOP monitoring, especially in high-risk groups.

## Introduction

One of the most common causes of permanent vision loss in adults around the world is glaucoma [1]. In glaucoma, a series of slowly progressing optic neuropathies result in the loss of retinal ganglion cells and their axons, leading to abnormalities in the visual field (VF) [2]. Glaucoma, which affects around 70 million people worldwide, is thought to be the main cause of permanent blindness [3].

Ocular hypertension (OHT) is a recognized risk factor for the onset of primary open-angle glaucoma (POAG), one of the main causes of blindness. However, not all OHT patients experience POAG, and not all POAG patients experience blindness, raising the question of whether OHT should be treated [4]. There has been discussion in recent years about how intraocular pressure (IOP) affects optic disc damage caused by glaucoma. Optic disc injury has been observed in eyes with an IOP within the “normal” range, the range of the IOP in the glaucomatous population is rather large. Therefore, it is difficult to identify an IOP level that will result in glaucomatous damage [5].

Several systemic and ocular risk factors may also influence the development of glaucoma. However, there are few known predisposing factors for OHT, and epidemiologic studies have shown mixed results on the association between OHT and purported risk factors [6].

In this study, we aimed to estimate the prevalence of OHT as a risk factor for glaucoma in Al-Baha City, Saudi Arabia, and to identify other possible risk factors associated with IOP elevation.

## Materials and methods

Study design and setting

A cross-sectional study was conducted to measure IOP and screen for glaucoma risk factors among middle-aged adults in Al-Baha City, Saudi Arabia. The study was conducted between January 2022 and June 2022.

Sampling and participants

The population of this study was recruited from residents aged 35 years or older in Al-Baha City. Exclusion criteria for this study were individuals younger than 35 years, those who submitted incomplete questionnaires, and those unable to provide informed consent for participation.

The sample size was determined using the statistical software Epi Info 7.2. The sample size for descriptive analysis was determined to be 40 participants, assuming a prevalence of OHT of 2.7% based on the existing literature [7]. A level of precision of 5% was chosen for the 95% confidence interval. The formula utilized to determine the sample size in this study was as follows: n=(PQ/L)2×(Zα/2)2, where P represents the prevalence of OHT, Q is equal to 1-P, L denotes the width of the confidence interval, and Z-score corresponding to the desired confidence level. The sample size was open to higher numbers to increase the accuracy of the results.

We used a non-probability consecutive sampling technique for recruiting participants. Individuals were selected from a glaucoma awareness campaign, which was organized at King Fahad Hospital in Al-Baha City.

Procedure and data collection

A written survey was given to the recruited participants to obtain demographic data and identify any risk factors for glaucoma. The participants were informed about the study's objectives and the confidentiality of the data. They consented to participate, completed the questionnaire, and underwent IOP measurements with a noncontact tonometer device (Shin-Nippon NCT-200) for IOP screening. OHT was defined in our study as cases where the IOP in the eyes exceeded 21 mmHg.

All researchers involved in this study also performed data entry. Following the verification process, the data were subsequently transferred directly to a statistics database.

Questionnaire

In this study, a reliable, pre-validated, and modified written questionnaire from a previous study was utilized. The questionnaire consisted of two sections. The initial section comprised questions pertaining to sociodemographic characteristics. The subsequent section consisted of a set of eight questions designed to identify risk factors associated with glaucoma.

Data analysis

Continuous data are presented as the mean and standard deviation, and categorical data are presented as the frequency. The chi-squared test was used to assess differences among the means for categorical variables. A P-value less than 0.05 was considered to be statistically significant for all statistical tests conducted.

Informed consent and ethical consideration

Informed consent was obtained from the participants prior to participation in the study. At the study onset, the participants were assured of the confidential nature of the patient-provider interaction. The investigators ensured confidentiality in data collection by assigning a code to replace patient identifiers (e.g., name). All collected data were kept secure, including hard and soft copies, with authorized access only. The research staff also maintained confidentiality and privacy. In addition, any personal information was not discussed during data analysis.

## Results

The study investigated the prevalence of OHT and risk factors for glaucoma in a middle-aged group of 111 participants. Table 1 outlines the participants' risk factors and demographic information. Mean IOP values for the right and left eyes according to different factors are listed in Tables 2-3, respectively. The findings indicated that age, gender, race, family history of glaucoma, past medical history, chronic medication use, eyedrop or ointment use, current eye issues, history of ophthalmic surgeries, and smoking history were associated with IOP changes.

**Table 1:** Demographic Profiles and Associated Risk Factors of Glaucoma in Study Participants

Factor	n	%
Age group		
35-39 years	30	27.0
40-44 years	18	16.2
45-49 years	24	21.6
50-55 years	21	18.9
>55 years	18	16.2
Gender		
Male	84	75.7
Female	27	24.3
Nationality		
Saudi	75	67.6
Non-Saudi	36	32.4
Family history of glaucoma		
No	102	91.9
Yes	9	8.1
Past medical history		
None	90	81.1
Vascular disease	12	10.8
Diabetes mellitus	9	8.1
Chronic medications use		
No	81	73.0
Yes	30	27.0
Eyedrops or ointment use		
No	75	67.6
Yes	36	32.4
Current eye issues		
No	57	51.4
Yes	54	48.6
History of ophthalmic surgeries		
No	93	83.8
Yes	18	16.2
History of smoking		
No	87	78.4
Yes	24	21.6
Race		
Asian	21	18.9
Caucasian	75	67.6
Black	15	13.5

**Table 2:** Comparative Mean Intraocular Pressure (IOP) Values in the Right Eye Across Different Independent Factors *A p-value <0.05 was considered statistically significant. IOP: intraocular pressure; SD: standard deviation

Factor	Mean IOP (mmHg) ± SD	p-value
Age group		0.446
35-39 years	18.6 ± 4.4	
40-44 years	19.4 ± 2.6	
45-49 years	20.1 ±3.8	
50-55 years	18.2 ± 2.9	
>55 years	19.4 ± 3.4	
Gender		0.451
Male	18.9 ± 3.3	
Female	19.6 ± 4.4	
Nationality		0.592
Saudi	19.2 ± 4.0	
Non-Saudi	18.8 ± 2.7	
Family history of glaucoma		0.206
No	19.0 ± 3.7	
Yes	20.6 ± 2.2	
Past medical history		0.046*
None	18.7 ± 3.5	
Vascular disease	21.5 ± 3.6	
Diabetes mellitus	19.7 ± 3.7	
Chronic medications use		0.597
No	19.0 ± 3.8	
Yes	19.4 ± 3.1	
Eyedrops or ointment use		0.083
No	19.5 ± 3.7	
Yes	18.2 ± 3.3	
Current eye issues		0.384
No	19.4 ± 4.1	
Yes	18.8 ± 3.1	
History of ophthalmic surgeries		<0.001*
No	19.9 ± 3.4	
Yes	15.1 ± 1.4	
History of smoking		0.051
No	18.7 ± 3.4	
Yes	20.4 ± 4.3	
Race		0.123
Asian	17.6 ± 2.2	
Caucasian	19.5 ± 4.0	
Black	19.2 ± 3.1	

**Table 3:** Comparative Mean Intraocular Pressure (IOP) Values in the Left Eye Across Different Independent Factors *A p-value <0.05 was considered statistically significant. IOP: intraocular pressure; SD: standard deviation

Factor	Mean IOP (mmHg) ± SD	p-value
Age group		0.502
35-39 years	19.1 ± 3.6	
40-44 years	20.9 ± 2.7	
45-49 years	20.2 ± 3.7	
50-55 years	19.5 ±2.4	
>55 years	19.8 ± 3.9	
Gender		0.150
Male	19.6 ± 3.1	
Female	20.6 ± 4.0	
Nationality		0.074
Saudi	20.2 ± 3.5	
Non-Saudi	19.0 ± 2.9	
Family history of glaucoma		0.068
No	19.7 ± 3.4	
Yes	21.8 ± 1.5	
Past medical history		0.029
None	19.4 ± 3.3	
Vascular disease	21.1 ± 2.9	
Diabetes mellitus	22.1 ± 3.1	
Chronic medications use		0.321
No	19.6 ± 3.4	
Yes	20.4 ± 3.2	
Eyedrops or ointment use		0.031*
No	20.3 ± 3.4	
Yes	18.9 ± 2.9	
Current eye issues		0.216
No	20.2 ± 3.5	
Yes	19.4 ± 3.1	
History of ophthalmic surgeries		<0.001*
No	20.6 ± 3.1	
Yes	16.0 ± 1.6	
History of smoking		0.008*
No	19.4 ± 3.3	
Yes	21.4 ± 3.1	
Race		0.002*
Asian	17.6 ± 2.2	
Caucasian	20.5 ± 3.5	
Black	19.7 ± 2.3	

Table 1 shows the demographic characteristics and risk factors of the participants. The study included 111 participants in different age groups ranging from 35-39 years to >55 years, comprising 84 (75.7%) males and 27 (24.3%) females, with 75 (67.6%) Saudi and 36 (32.4%) non-Saudi participants. Additionally, there were nine (8.1%) participants with and 102 (91.9%) without a family history of glaucoma. Data on previous medical history included participants with no history 90 (81.1%), those with vascular disease 12 (10.8%), and those with diabetes mellitus 9 (8.1%). Furthermore, 30 (27%) participants used chronic medications, while 81 (73%) did not. A total of 36 (32.4%) participants used eyedrops or ointments, and 75 (67.6%) did not. Participants with current eye issues accounted for 48.6% (54), without for 51.4% (57); with a history of ophthalmic surgeries for 16.2% (18), without for 83.8% (93); and with a history of smoking for 21.6% (24), without for 78.4% (87). Table 1 also shows the number and percentage of participants according to race, including Asian 21 (18.9%), Caucasian 75 (67.6%), and Black 15 (13.5%).

Table 2 shows the mean IOP of the right eye of the participants according to different factors. The mean IOP of the right eye ranged from 15.1 ± 1.4 mmHg to 21.5 ± 3.6 mmHg in different subgroups. For the age group of 35-39 years, the mean IOP was 18.6 ± 4.4 mmHg, and for those aged 45-49 years, it was 20.1 ± 3.8 mmHg. Males had a mean IOP of 18.9 ± 3.3 mmHg, while females had a mean IOP of 19.6 ± 4.4 mmHg. Non-Saudi participants had a mean IOP of 19.2 ± 4.0 mmHg, whereas Saudi participants had a mean IOP of 18.8 ± 2.7 mmHg. Participants with a family history of glaucoma had a mean IOP of 20.6 ± 2.2 mmHg, while those without a family history had a mean IOP of 19.0 ± 3.7 mmHg. Participants with a history of ophthalmic surgeries had a mean IOP of 15.1 ± 1.4 mmHg, compared to 19.9 ± 3.4 mmHg for those without such history. Those without any past medical history had a mean IOP of 18.7 ± 3.5 mmHg. Participants with a history of vascular disease had a mean IOP of 21.5 ± 3.6 mmHg, and those with diabetes mellitus had a mean IOP of 19.7 ± 3.7 mmHg.

Among participants not taking chronic medication, the mean IOP was 19.0 ± 3.8 mmHg, while for those on chronic medication, it was 19.4 ± 3.1 mmHg. Those not using eyedrops or ointments had a mean IOP of 19.5 ± 3.7 mmHg, in contrast to 18.2 ± 3.3 mmHg for those who did use them. Participants without current eye issues had a mean IOP of 19.4 ± 4.1 mmHg, and those with current eye issues had a mean IOP of 18.8 ± 3.1 mmHg. Smokers had a higher mean IOP of 20.4 ± 4.3 mmHg compared to non-smokers, who had a mean IOP of 18.7 ± 3.4 mmHg. Among the participants, Caucasian individuals had a mean IOP of 19.5 ± 4.0 mmHg, Asians had the lowest mean IOP of 17.6 ± 2.2 mmHg, and Black participants had a mean IOP of 19.2 ± 3.1 mmHg.

Table 3 presents the mean IOP for the left eye of participants, categorized by different factors. The mean IOP values ranged from 19.1 ± 3.6 mmHg to 20.2 ± 3.7 mmHg across various age groups, demonstrating variability with age. Specifically, the mean IOP was 19.1 ± 3.6 mmHg for those aged 35-39 years, 20.9 ± 2.7 mmHg for the 40-44 years group, 20.2 ± 3.7 mmHg for 45-49 years, 19.5 ± 2.4 mmHg for 50-55 years, and 19.8 ± 3.9 mmHg for participants over 55 years.

Gender differences in mean IOP were evident, with males having a mean IOP of 19.6 ± 3.1 mmHg and females showing a mean IOP of 20.6 ± 4.0 mmHg. Among the study population, non-Saudi participants had a mean IOP of 19.0 ± 2.9 mmHg, while Saudi participants had a slightly higher mean IOP of 20.2 ± 3.1 mmHg. A considerable increase in mean IOP was noted in participants with a family history of glaucoma, having a mean IOP of 21.8 ± 1.5 mmHg, compared to those without such a history who had a mean IOP of 19.7 ± 3.4 mmHg.

The study also examined the mean IOP in relation to participants' medical history. Those with a history of ophthalmic surgeries had a mean IOP of 16.0 ± 1.6 mmHg, in contrast to 20.6 ± 3.1 mmHg for those without a surgery history. Participants with no past medical history had a mean IOP of 19.4 ± 3.3 mmHg, whereas individuals with the vascular disease had a mean IOP of 21.1 ± 2.9 mmHg and those with diabetes mellitus had a mean IOP of 22.1 ± 3.1 mmHg.

Regarding medication use, participants not on chronic medications had a mean IOP of 19.6 ± 3.4 mmHg, whereas those on chronic medication had a mean IOP of 20.4 ± 3.2 mmHg. The use of eyedrops or ointments was associated with a mean IOP of 18.9 ± 2.9 mmHg, which was lower than the mean IOP of 20.3 ± 3.4 mmHg for those not using them. Additionally, participants without current eye issues had a mean IOP of 20.2 ± 3.5 mmHg, whereas those with current eye issues had a mean IOP of 19.4 ± 3.3 mmHg.

Lastly, lifestyle and racial background were also reflected in the mean IOP values. Smokers had a mean IOP of 21.4 ± 3.1 mmHg, while non-smokers had a mean IOP of 18.7 ± 3.4 mmHg. Mean IOP varied among different ethnicities, with Caucasian participants having a mean IOP of 20.5 ± 3.5 mmHg, Asians having the lowest mean IOP at 17.6 ± 2.2 mmHg, and Black participants having a mean IOP of 19.7 ± 2.3 mmHg.

## Discussion

One of the primary causes of permanent vision loss is glaucoma [8,9]. It is estimated that glaucoma affects 3.5% of people in the world between the ages of 40 and 80 years. Furthermore, 111.8 million individuals are predicted to develop glaucoma in 2040 due to the increasing number and percentage of elderly people in the population [10]. Glaucoma has been reported to be one of the three leading causes of visual impairment in Saudi Arabia, accounting for 5.7% of all cases [11,12].

Multiple factors make glaucoma a public health issue [13]. First, disease diagnosis and treatment are difficult as some forms are asymptomatic. Patients will not notice vision loss until the optic nerve fibers and VFs are damaged extensively [14]. In angle-closure glaucoma, the damage progresses rapidly toward blindness and is hard to slow down or manage. Second, reading, driving, and movement can be affected by glaucoma [15]. Glaucoma patients have reduced life quality due to functional impairments. Third, advanced glaucoma therapy is costly, which could impose a large economic burden on low-vision glaucoma patients [15]. Due to these factors, a better understanding of the disease frequency and distribution pattern would facilitate disease treatment and glaucoma prevention.

We did not observe a significant association between age and IOP for the left or right eye. Similarly, a family-based longitudinal study in Korea and Mongolia tracked 3096 patients for more than 10 years and found no association between IOP and older age [16]. There is debate on how aging affects the IOP. Previous cross-sectional studies in America [17,18] and Italy [19] revealed a substantial positive association between age and IOP; however, investigations in Europe, Japan, Korea, and other countries observed the opposite effect [20-27].

A study carried out in Europe found a U-shaped relationship between age and IOP and concluded that this relationship may explain the contrasting findings from different studies [28]. The equilibrium between aqueous fluid production and resistance in both conventional and non-conventional outflow routes determines the IOP [29-31]. Given that aging has been shown to reduce the formation of the aqueous humor, the mechanism underlying the effects of aging on the IOP may not be clear-cut. The inconsistent results among studies may be attributed to regional variations in eye characteristics, such as the incidence of myopia [32,33].

Based on the existing literature, the link between IOP and sex is not clearly established. In our study, there was no evidence of a significant relationship between sex and IOP (P=0.451 for the right eye and 0.150 for the left eye). These findings are consistent with those of several studies [22,23,27,34]. However, a number of studies observed a greater IOP among females than among males [35-38], and vice versa [19,24,39]. Although it is unclear why there are gender-specific variations in the IOP, they have been attributed to menopausal hormone changes and the effects of IOP-lowering medications such as estrogen and nitric oxide. In addition, the observed variations in IOP may be attributed to the interplay of several systemic factors. These include systolic blood pressure, body mass index, regional and ethnic variations, and overall physical health [40].

In our study, past medical history and IOP were significantly correlated (P=0.046). Several studies have reported a significant correlation between high blood pressure and IOP [16,21,23-25,27,34,41-44]. In IOP elevation, higher blood pressure has been proposed to cause an increase in the filtration fraction of the aqueous humor via an increase in ciliary artery pressure, and increased IOP is the consequence of elevated serum corticoids and sympathetic tone [45,46]. Additionally, nocturnal hypotension has been identified as a significant risk factor for VF loss in normal-tension glaucoma (NTG), with prolonged periods of mean arterial pressure (MAP) being 10 mmHg below daytime levels predicting VF progression [47]. Furthermore, peripheral vasospasm, evidenced by greater decreases in finger temperature post-cold pressor test, has been associated with accelerated VF progression in NTG patients, independent of IOP levels [48]. Lastly, the role of retinal blood flow autoregulation in relation to IOP and blood pressure variations suggests that individuals' capacity to regulate retinal hemodynamics is contingent on their BP and autoregulatory abilities, which could explain inconsistencies in the clinical literature about the relationship between IOP and retinal hemodynamics [49]. On the other hand, a number of studies have linked glaucoma to decreased ocular perfusion pressure [50,51]. These results demonstrate the diverse relationships and complexity of glaucoma. Although a positive association between systemic blood pressure and IOP has been established, further research is necessary to determine the relationship between systemic blood pressure and glaucoma.

Various studies have also shown a strong association between diabetes and an increase in the IOP [20,21,23-25,27,34,52]. However, several studies indicated no conclusive link [43,53]. It has been suggested that the osmotic gradient induced by hyperglycemia, which forces the aqueous humor into the anterior chamber, is the cause of the increased IOP in diabetic patients [54]. Additionally, diabetes mellitus may induce glaucoma and other eye diseases via vascular damage, independent of an increase in the IOP [55].

Our findings showed that smoking had no apparent association with IOP, consistent with some studies [56,57] but not others [27,50,53]. These inconsistent findings might be attributed to the different research designs, the size of the population under examination, or the various ways in which smoking was defined for the purpose of each study. Our findings demonstrated that there was no association between current eye conditions and IOP, which is in agreement with some studies [53,58,59] but contradicts other studies [60,61]. We also found a significant association between previous eye operations and IOP. Additionally, there was a strong correlation between race and IOP.

Our study has some limitations, including the use of a noncontact tonometer, which is generally considered to be less accurate than a Goldmann applanation tonometer. Additionally, there were no measurements of the central corneal thickness, which is known to contribute to tonometry measurement problems. One other possible drawback is that this study's approach was cross-sectional instead of longitudinal.

## Conclusions

This study highlights the prevalence of OHT among middle-aged adults, with significant variations observed across gender and nationality. While factors like family history of glaucoma, vascular diseases, diabetes mellitus, and chronic medication use were found to be associated with OHT, our study did not observe a significant association with smoking or age.
